# Psychosocial and behavioral outcomes in the adult workforce during the COVID-19 pandemic: a 1-Year longitudinal survey

**DOI:** 10.1186/s12889-023-15536-8

**Published:** 2023-04-03

**Authors:** Araliya M. Senerat, Zachary C. Pope, Sarah A. Rydell, Aidan F. Mullan, Véronique L. Roger, Mark A. Pereira

**Affiliations:** 1 Formerly Well Living Lab, 221 1st Ave SW, Rochester, MN 55902, 507-550-1139 x664 USA; 2 Now with Urban Health Collaborative, Dornsife School of Public Health, Drexel University, 3600 Market Street, 7th Floor, Philadelphia, PA 19104 USA; 3 Department of Physiology and Biomedical Engineering, Mayo Clinic, 200 1st St SW, Rochester, MN 55905 USA; 4 Division of Epidemiology & Community Health, School of Public Health, University of Minnesota, 1300 South Second Street, Suite 300, Minneapolis, MN 55454 USA; 5 Department of Quantitative Health Sciences, Mayo Clinic, Rochester, MN USA; 6 Formerly Department of Cardiovascular Diseases, College of Medicine, Mayo Clinic, Rochester, MN USA; 7 Now with Epidemiology and Community Health Branch, National Heart, Lung and Blood Institute, National Institutes of Health, Bethesda, USA; 8 Well Living Lab, 221 1st Ave. SW, Suite 100, Rochester, MN 55902 USA

**Keywords:** COVID-19, Psychosocial health, Behavioral health, Longitudinal study

## Abstract

**Background:**

Efforts to limit the spread of COVID-19 have included public space closures, mask usage, and quarantining. Studies regarding the impact of these measures on the psychosocial and behavioral health outcomes of the workforce have focused frequently on healthcare employees. To expand the literature base, we deployed a one-year longitudinal survey among mostly non-healthcare employees assessing changes in select psychosocial outcomes, health behaviors, and COVID-19-related transmission prevention behaviors and perceptions.

**Methods:**

We deployed the CAPTURE baseline survey across eight companies from November 20, 2020-February 8, 2021. The baseline survey included questions on psychosocial outcomes, health behaviors, and COVID-19 transmission prevention behaviors, with several questions containing a retrospective component to cover the time period prior to the pandemic. Additional questions on vaccination status and social support were subsequently added, and the updated survey deployed to the same baseline participants at three, six, and 12 months after baseline survey deployment. We analyzed data descriptively and performed Friedman’s and subsequent Wilcoxon-signed rank tests, as appropriate, to compare data within and between time points.

**Results:**

A total of 3607, 1788, 1545, and 1687 employees completed the baseline, 3-month, 6-month, and 12-month CAPTURE surveys, respectively, with 816 employees completing all four time points. Employees reported higher stress, anxiety, fatigue, and feelings of being unsafe across all time points compared to pre-pandemic. Time spent sleeping increased initially but returned to pre-pandemic levels at follow-up. Lower rates of physical activity and higher rates of non-work screen time and alcohol consumption relative to pre-pandemic were also reported. Over 90% of employees perceived wearing a mask, physical distancing, and receiving the COVID-19 vaccine as ‘moderately’ or ‘very important’ in preventing the spread of COVID-19 across all time points.

**Conclusions:**

Relative to pre-pandemic, poorer psychosocial outcomes and worsened health behaviors were observed across all time points, with values worse at the baseline and 12-month time points when COVID-19 surges were highest. While COVID-19 prevention behaviors were consistently deemed to be important by employees, the psychosocial outcome and health behavior data suggest the potential for harmful long-term effects of the pandemic on the well-being of non-healthcare employees.

**Supplementary Information:**

The online version contains supplementary material available at 10.1186/s12889-023-15536-8.

## Background

Since March 2020, coronavirus disease-2019 (COVID-19) has infected > 500 million individuals and led to > 6 million deaths. Efforts to limit the spread of severe acute respiratory syndrome coronavirus-2 (SARS-CoV-2), the virus causing COVID-19, have included public space closures and significant changes to daily routines. While well intended, these changes may have had adverse psychosocial and behavioral effects on the population. Among adults, a recent study observed notable perceived deterioration in mental health during the COVID-19 pandemic [1], with a meta-analysis noting that fear of COVID-19 had a significant association with depression, anxiety, and stress [2]. These findings are particularly concerning when mapped onto pre-existing work-related stressors among the adult workforce.

Studies regarding the impact of COVID-19 on the workforce have primarily focused on healthcare employees [3]. Comparatively fewer studies have been conducted among non-healthcare employees. For many non-healthcare employees, the move to work from home (WFH) has presented unique challenges, particularly among those with childcare responsibilities and/or those struggling to cope with social isolation. Among the scant literature on non-healthcare employees, Xiao et al. [4] noted poorer overall mental well-being to be associated with several factors including adjusted work hours, children at home while working, more work distractions, and less communication with coworkers. Similarly, our prior research from earlier in the pandemic found non-healthcare employees perceived higher stress, anxiety, fatigue, feelings of being unsafe, lack of companionship, and feelings of isolation relative to their pre-pandemic state [5].

Health behaviors have also been negatively impacted by the pandemic. A systematic review reported that, generally, physical activity was lower and sedentary behavior higher among healthy adults during the initial 2020 COVID-19 lockdown versus pre-pandemic [6]. Another study observed not only lower physical activity over this time period but that participants also perceived greater appetite and unhealthy eating behaviors [1]. Sleep problems and more screen time have also been observed throughout the pandemic relative to pre-pandemic [7–9], with Chen et al. [9] noting higher engagement in alcohol consumption and smoking. Our own research among non-healthcare employees aligns with these observations [5]. Poorer health behaviors are important to consider given their impact on risk for obesity, diabetes, cardiovascular disease, and adverse mental health outcomes [10–13]. Such potential outcomes may have deleterious effects on companies through increased employee absenteeism and turnover and reduced employee well-being, productivity, and satisfaction [14, 15]. Finally, employees’ COVID-19-related transmission prevention behaviors are also critical to assess in a simultaneous manner. For example, non-compliance with transmission prevention behaviors (e.g., physical distancing, mask wearing, etc.) may increase the likelihood and ultimate acquisition of a COVID-19 infection [16] and result in higher employee absenteeism, lower productivity, and poorer ability to engage in health behaviors. Alternatively, the psychosocial aspects of engaging in transmission prevention behaviors cannot be overlooked, as these same behaviors may lead to poorer psychosocial outcomes (e.g., greater feelings of social isolation) [17].

Of the few COVID-19-related non-healthcare employee studies to date, most have been cross-sectional. We therefore conducted a one-year study among largely non-healthcare employees, with a primary aim to assess changes in select psychosocial outcomes and health behaviors at baseline and three, six, and 12 months. A secondary aim was to assess COVID-19-related transmission prevention behaviors and perceptions.

## Methods

This study was approved by the University of Minnesota (IRB #: STUDY00010426) and Mayo Clinic (IRB #: 20-007642) Institutional Review Boards. All participants gave informed consent at each survey time point. All procedures were performed in accordance with the ethical standards of the institution and/or national research committee and with the 1964 Helsinki Declaration and its later amendments or comparable ethical standards [18]. Further, we used the Strengthening the Reporting of Observational Studies in Epidemiology (STROBE) guidelines [19] to guide our descriptions of this study (Supplementary Material).

### Study Design and Survey Deployment

We developed a survey titled, “*Characterizing Awareness of SARS-CoV-2 PrevenTion and Understanding Responses and Experiences (CAPTURE) Survey*”, to collect information on psychosocial outcomes and COVID-19 transmission prevention behaviors in the workplace as well as overall health behaviors. The CAPTURE baseline survey (*baseline*) consisted of 48 questions across eight sections and was deployed from November 20, 2020 to February 8, 2021. Results from the CAPTURE baseline survey have been published [5]. For the CAPTURE 3-month survey (*3-mo*), we added a question on vaccination status before deployment from March 8, 2021 to May 6, 2021, bringing the total number of questions to 49. We included one additional question regarding social support on the CAPTURE 6-month (*6-mo*; deployed May 20, 2021 to August 4, 2021) and 12-month (*12-mo*; deployed November 29, 2021 to February 7, 2022) surveys, for 50 total questions. We have included a copy of the CAPTURE 12-mo survey in the Supplementary Material and reviewed its development in ‘Measures’.

Surveys were deployed using Mayo Clinic’s Qualtrics Platform to eight companies that originally agreed to participate in the CAPTURE baseline survey, with the 3-mo, 6-mo, and 12-mo surveys deployed only to employees that provided contact information and consented to the baseline survey. For seven companies, 3-mo, 6-mo, and 12-mo survey emails were sent from Mayo Clinic’s Qualtrics Platform. For the remaining company, an anonymous survey link was sent out to all employees in the company at this company’s request. Survey responses were tracked using emails provided at baseline and then deidentified, with employees given a unique study ID at baseline that consisted of four random numbers. We used this ID to track each individual employee at all later time points.

### Inclusion and exclusion criteria

Inclusion and exclusion criteria at the company- and employee-level remained identical across all time points, with expanded details in our prior publication [5]. Briefly, we included U.S.-based companies that agreed to their employees participating in the CAPTURE Survey and queried employees who were ≥ 18 years old, English speaking, and employed by the company at the time of deployment. Employee-level inclusion criteria also included spending ≥ 50% of their workweek working indoors given the higher transmission risk of SARS-CoV-2 while indoors.

### Measures

Questions from the baseline survey stayed consistent for the 3-mo, 6-mo, and 12-mo surveys, with four questions (Q19, Q21, Q23, & Q36a) edited to take into consideration changes in the COVID-19 pandemic (e.g., arrival of vaccines). Briefly, CAPTURE Survey questions asked employees to report demographic characteristics and the impact of the COVID-19 pandemic on socioeconomic factors, psychosocial outcomes (e.g., stress, anxiety, fatigue, perceived productivity), and health behaviors (e.g., physical activity, screen-time, alcohol consumption). Employees also reported on COVID-19-related transmission prevention behavior engagement (e.g., masking, physical distancing) and perceptions. All questions were validated and drawn from the literature [20–25] and by consulting researchers completing similar investigations on COVID-19-related outcomes, with detailed information on all questionnaires used within the CAPTURE Survey available in our baseline CAPTURE Survey publication [5]. The four additional questions added to the follow-up CAPTURE survey deployments are described below.

#### COVID-19 vaccination status and vaccine-related perceptions

Questions on COVID-19 vaccination status were added to the 3-mo, 6-mo, and 12-mo surveys. Employees were asked about the type and date of each vaccine received; if the vaccine type was not listed, employees could enter the name of the vaccination they received. For the 12-mo survey, these four follow-up questions were edited to take into consideration the one-shot Johnson & Johnson vaccine.

Two statements were added to investigate whether an employee’s company had been: *“Encouraging staff to get vaccinated”* and “*Sponsoring vaccination efforts”.* Further, one question was added on how important employees perceived *“Getting the COVID-19 vaccine”* was in preventing the spread of COVID-19, with employees also asked whether they agreed or disagreed with the following statement: *“Getting the COVID-19 vaccine is very important to me”*. All four statements were present in the CAPTURE 3-mo, 6-mo, and 12-mo surveys.

Questions regarding COVID-19 vaccination status and vaccine-related perceptions were developed and deployed by our research team after discussion with other researchers also completing COVID-19-related research as well as internal vetting by our research team.

#### Social support

For the CAPTURE 6-mo and 12-mo surveys, the validated NHANES Social Support Questionnaire [26] was added to better understand the association between social support and psychosocial responses during the pandemic. The questions asked employees if they can count on anyone to provide emotional support, if they could have used more emotional support than received, and, if so, how much more.

### Recruitment

More details on recruitment can be found in our prior CAPTURE Survey publication [5]. We conducted an extensive company recruitment campaign of 234 U.S.-based companies. After discussions with 18 companies regarding CAPTURE Survey involvement, eight companies agreed to participate. No requirement was made by any participating company that employees participate, as the CAPTURE survey was entirely voluntary.

### Statistical analyses

Survey responses for all time points were uploaded to a secure database from Qualtrics. Data were cleaned and analyzed using Microsoft Excel and Python 3.9 Jupyter Notebook for Windows 1.3.1093. Duplicates at any time point were dropped based on the date of the first survey completion.

Data from all time points were analyzed separately, with descriptive analyses performed; main results presented herein are part of the “full dataset” containing all responses from any employee that completed the CAPTURE Survey at each time point. We collapsed the following response categories when analyzing results from 5-point Likert type scales: (1) ‘never’ and ‘rarely’; and (2) ‘quite a bit’ and ‘all the time’. Given that we did not augment the ‘moderate’ response, collapsing these response categories created three distinct levels during analysis. Using national guidelines [27–29], we also collapsed responses regarding duration of physical activity/day (> 30 min/day vs. <30 min/day), hours of sleep/night (> 7 h/night vs. ≤7 h/night), and drinks/week (0 drinks/week, 1–6 drinks/week, 7–15 drinks/week, or ≥ 16 drinks/week), with non-work screen time collapsed to < 2 h/day and > 2 h/day based on literature noting the detrimental health effects of > 2 h of non-work screen time [30–32].

After performing descriptive analyses on the full dataset, we then performed sensitivity analyses on a “completers dataset” by merging all time points containing only employees who completed all four survey time points. We first completed descriptive analyses on the completers dataset. Next, we used the Friedman’s test to assess whether any difference existed between time points for our psychosocial and behavioral outcome measures. This test was chosen given that it represents the non-parametric version of an ANOVA and best fit the 1) longitudinal nature of the study and 2) distribution of data being collected. When the Friedman’s test was significant, we then performed Wilcoxon-signed rank tests to discern where differences between any two specific time points were present. The p-values for our Wilcoxon-signed rank tests were adjusted for multiple comparisons using the Benjamini-Hochberg correction.

Importantly, regardless of the dataset being analyzed, it is notable that some CAPTURE baseline survey questions had a retrospective component. As one example, employees were asked about their stress levels ‘before the COVID-19 pandemic’ and ‘now, during the COVID-19 pandemic’ during the baseline survey. Therefore, these results are presented as two time points: “pre-pandemic” [before COVID-19] and “baseline” [during COVID-19].

## Results

In total, 3607, 1788, 1545, and 1687 employees across 8 companies completed the baseline, 3-mo, 6-mo, and 12-mo CAPTURE surveys, respectively, with response rates for the latter three survey time points being 49.6%, 42.8%, and 46.8%. These data comprised the full dataset. A total of 816 employees across these companies completed all four survey time points and comprised the completers dataset.

### Demographics

**Fig. 1:**
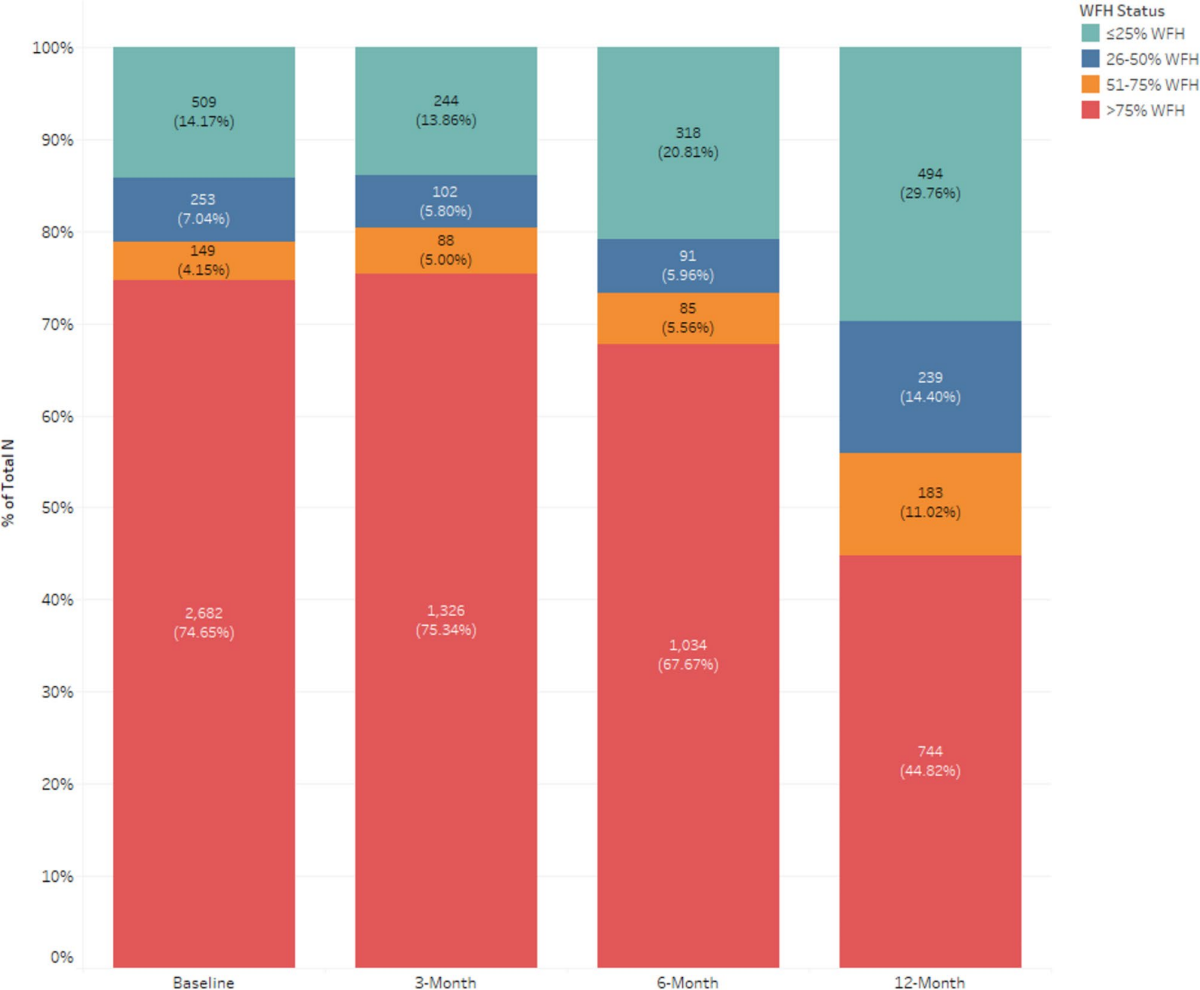
Work from Home (WFH) Categories for the Full Dataset during the COVID-19 Pandemic

Demographics for the full and completers dataset can be found in Table 1. Most employees were from the Midwest and well educated. The majority stated their health was in ‘good’, ‘very good’, or ‘excellent’ condition (baseline: 91.7%; 3-mo: 90.8%; 6-mo: 91.9%; 12-mo: 91.7%). Figure 1 shows the WFH status of employees at each time point stratified into four categories: ≤25%, 26–50%, 51–75%, and > 75%. At baseline, 80.4% of employees stated they ‘worked remotely or from home’. This trended lower throughout follow-up. At 12-mo, 31.7% of employees stated they were ‘working a combination model (part-time at home and part-time at the office)’, mirroring societal trends and the “hybrid work model”.

**Table 1:** Demographic Results for the Full Dataset and Completers Dataset*

Characteristic	Full Dataset (N (%))				Completers Dataset (N (%))				
	**Baseline**	**3-Mo**	**6-Mo**	**12-Mo**	**Baseline**	**3-Mo**	**6-Mo**	**12-Mo**	
**N**	3607	1788	1545	1687	816				
**Gender**									
Female/Woman	2292 (67.4)	1235 (71.8)	1077 (73.0)	1169 (72.3)	591 (73.8)	587 (73.4)	579 (73.3)	580 (73.3)	
Male/Man	1063 (31.3)	461 (26.8)	377 (25.5)	414 (25.6)	202 (25.2)	200 (25.0)	199 (25.2)	193 (24.4)	
Other/Nonbinary	34 (1.0)	18 (1.1)	18 (1.2)	24 (1.5)	8 (1.0)	3 (0.4)	10 (1.3)	12 (1.5)	
**Age**									
< 35	896 (26.9)	447 (26.3)	346 (23.7)	349 (21.7)	209 (26.4)	198 (24.9)	187 (23.9)	184 (23.4)	
35–65	2309 (69.3)	1201 (70.5)	1056 (72.1)	1190 (74.1)	560 (70.8)	577 (72.6)	571 (72.9)	575 (73.2)	
> 65	125 (3.8)	55 (3.2)	62 (4.2)	67 (4.2)	22 (2.8)	20 (2.5)	25 (3.2)	27 (3.4)	
**Hispanic or Latino/Latina/Latinx**	124 (3.5)	51 (3.0)	45 (3.1)	34 (2.1)	19 (2.4)	19 (2.4)	19 (2.4)	20 (2.5)	
**Which of the following best describes you?**									
Asian, Black, or African-American	217 (6.4)	74 (4.3)	53 (3.6)	56 (3.5)	28 (3.5)	27 (3.38)	30 (3.8)	27 (3.4)	
Other	216 (6.4)	90 (5.3)	82 (5.6)	78 (4.8)	40 (5.0)	39 (4.9)	40 (5.1)	39 (4.9)	
White	2924 (86.0)	1537 (89.4)	1328 (90.0)	1466 (90.6)	726 (91.4)	724 (90.5)	710 (89.9)	717 (90.6)	
**Current marital status**									
Married or partnered	2469 (72.6)	1242 (72.2)	1082 (73.4)	1186 (73.4)	590 (73.7)	581 (72.6)	579 (73.3)	576 (72.9)	
**Highest level of schooling completed**									
Less than Bachelor’s degree	364 (10.7)	138 (8.0)	130 (8.8)	149 (9.2)	58 (7.2)	58 (7.3)	55 (7.0)	59 (7.5)	
Bachelor’s degree	1260 (37.1)	653 (38.0)	545 (37.0)	580 (35.8)	290 (36.2)	291 (36.4)	287 (36.3)	290 (36.7)	
Master’s, Professional, or Doctoral degree	1762 (51.8)	924 (53.7)	796 (54.0)	882 (54.5)	453 (56.6)	448 (56.0)	445 (56.3)	440 (55.6)	

*‘Prefer not to answer’ responses were not included in the table and were less than 2% of responses

### Psychosocial outcomes

**Fig. 2:**
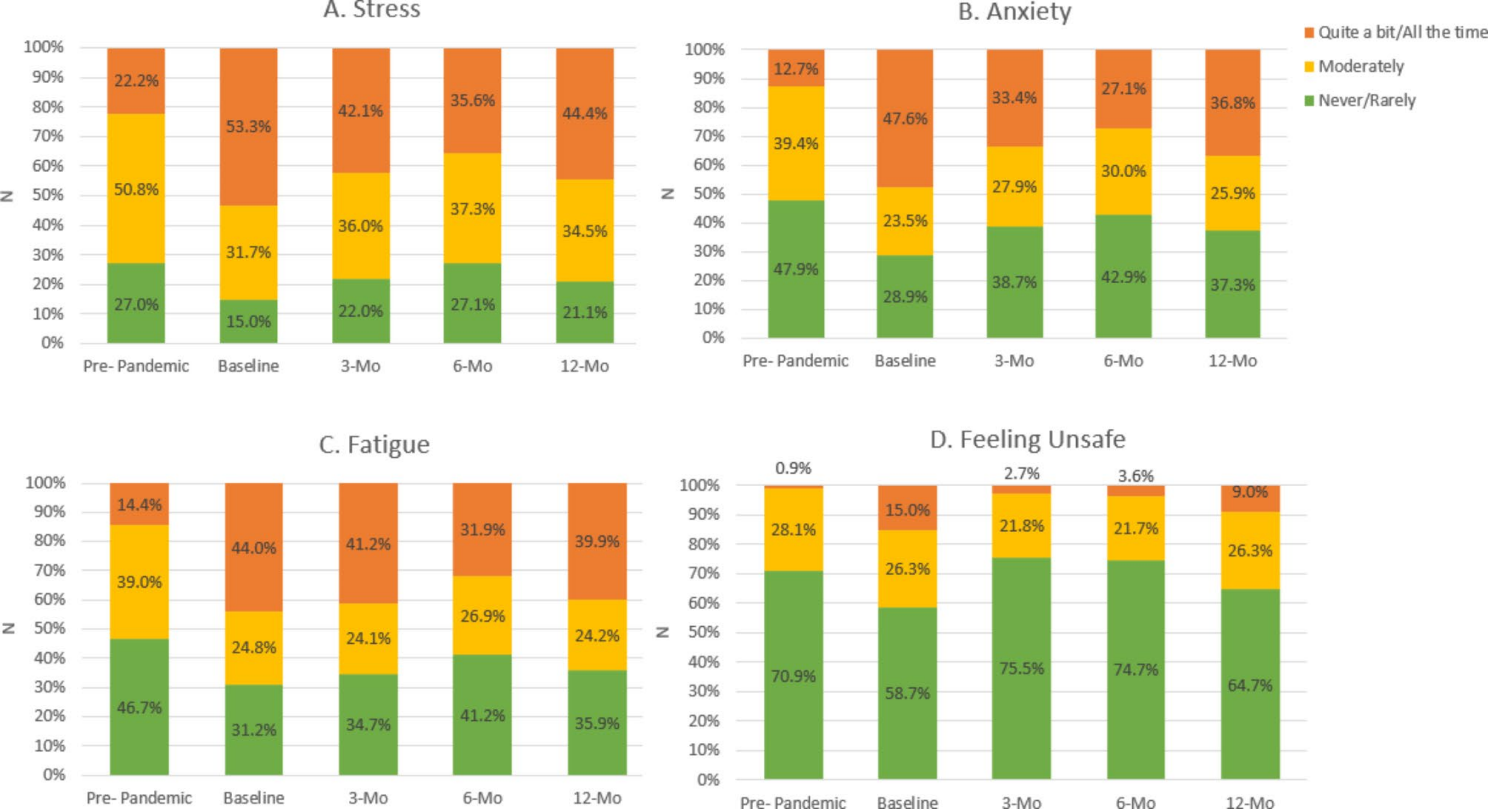
Psychosocial Responses for the Full Dataset Across Time Points*. (*Responses for ‘Prefer not to answer’ are not shown here. Sample size for each time point differ and are reported under ‘Results’)

Figure 2a-d shows employees’ reported stress, anxiety, fatigue, and feelings of being unsafe while completing work-related duties across all time points from the full dataset. Stress, anxiety, and fatigue demonstrated similar trends wherein employees experienced markedly higher prevalence of these feelings at baseline versus pre-pandemic, with these feelings persisting during the remaining time points (see Fig. 2a-d). While most employees reported ‘never’ or ‘rarely’ feeling unsafe across all time points, a notably higher prevalence of feeling unsafe ‘quite a bit’ or ‘all the time’ was observed at baseline (15.0%) and 12 months (9.0%) compared to the 3-mo and 6-mo time points. As shown in Supplemental Fig. 1 in Additional File #3, employees also reported higher prevalence of feeling a lack of companionship and more isolation at baseline versus pre-pandemic, with these feelings persisting throughout follow-up. Largely similar trends were seen for feelings of being left out. Further, while most employees stated they had someone that could provide emotional support during COVID-19, nearly half reported they could have used more emotional support than received. Analyses of the completers dataset showed similar trends, with significant changes and differences between time points (Supplemental Table 1 in the Supplementary Material).

### Behavioral outcomes

**Fig. 3:**
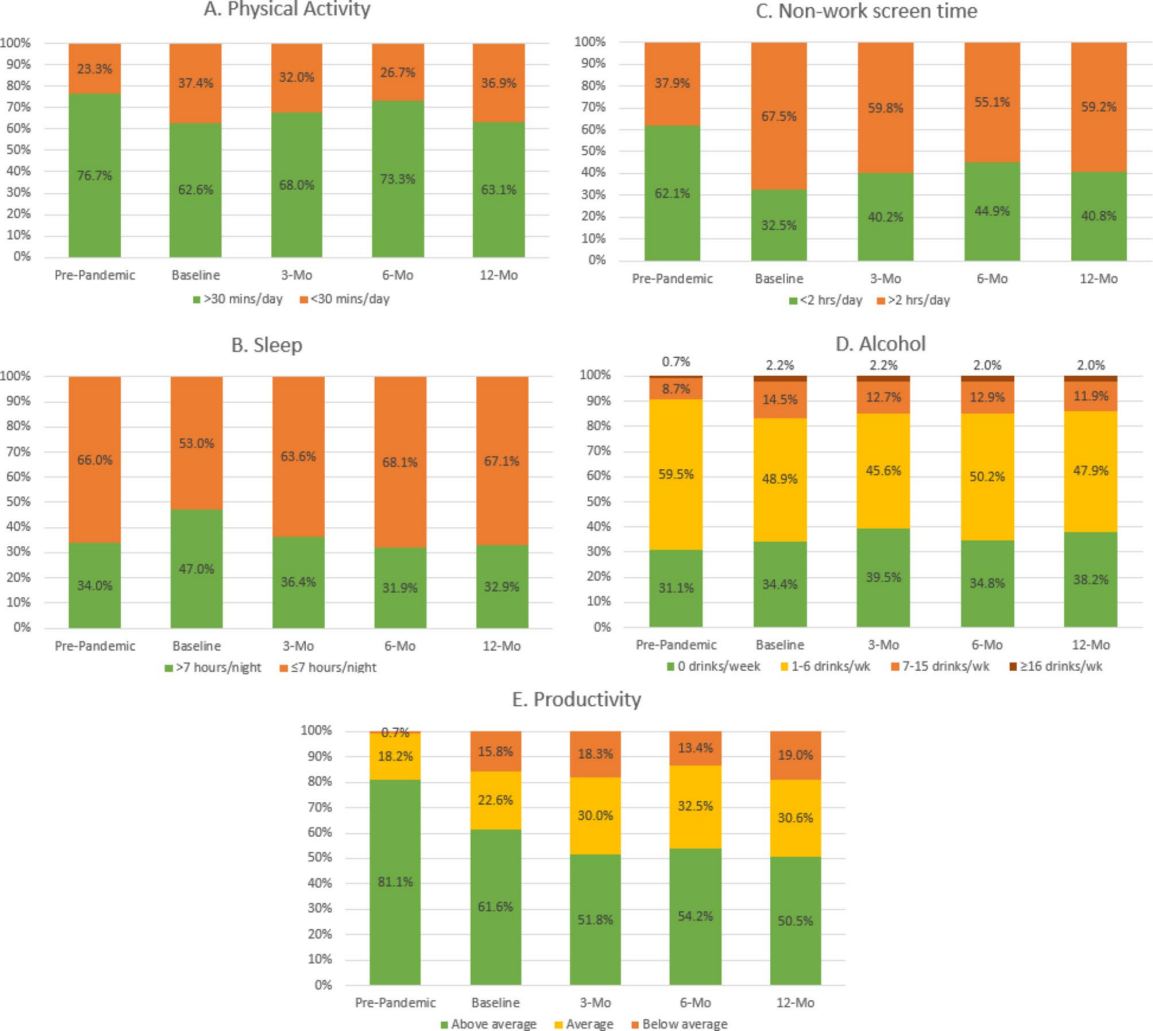
Behavioral Responses of the Full Dataset Across Time Points*. (*Responses for ‘Prefer not to answer’ are not shown here. Sample size for each time point differ and are reported under ‘Results’)

Figure 3a-e displays results for health behaviors. Pre-pandemic, 76.4% of employees reported > 30 min of physical activity, with physical activity participation at this duration trending lower at all subsequent time points. For sleep, 34.2% employees reported sleeping > 7 h/night pre-pandemic. At baseline, the percentage of employees sleeping > 7 h/night was higher, but this trend did not hold, with sleep duration returning to pre-pandemic levels thereafter. Non-work screen time was relatively low pre-pandemic, with 37.7% of employees reporting > 2 h/day. At baseline, however, this trend reversed, with 67.5% reporting > 2 h/day of non-work screen time and all subsequent time points remaining above 50% for this duration cutoff. Alcohol consumption appeared to have similar trends. Pre-pandemic, 9.3% of employees reported ≥ 7 drinks/week. Prevalence of ≥ 7 drinks/week was higher at every time point thereafter (baseline: 16.5%; 3-mo: 14.7%; 6-mo: 14.8%; and 12-mo: 13.8%). Finally, employees reported ‘above average’ or ‘high’ productivity at pre-pandemic but this trended lower at baseline (80.4–61.4%), with levels remaining lower than pre-pandemic levels thereafter. Analyses of the completers dataset showed similar trends, with changes and differences between time points significant (Supplemental Table 2 in the Supplementary Material).

### COVID-19-Related Prevention behaviors and perceptions

Across all time points, most employees perceived mask wearing, physical distancing, and getting vaccinated as ‘very important’ in preventing the spread of COVID-19. By the 12-month time point, 98.6% of respondents had received at least an initial two- or one-shot vaccination regimen. Employees reported the highest prevalence of ‘agreeing’ or ‘strongly agreeing’ to feeling threatened by COVID-19 or feeling stressed around other people due to risk of exposure to the virus at baseline, with this level of agreement decreasing at subsequent time points. When asked to what extent they viewed COVID-19 as having a ‘positive’ or ‘negative’ impact on their work, 68.0% stated ‘negative’ at baseline, which trended lower at 3-mo (62.8%) and 6-mo (56.3%). However, 70.7% of employees stated a ‘negative’ impact at 12-mo. The above results and additional COVID-19-related observations can be found in Supplemental Tables 3–6 in the Supplementary Material.

## Discussion

We sought to assess longitudinal changes in select psychosocial outcomes and health behaviors during the COVID-19 pandemic among non-healthcare employees, with a secondary aim being the examination of COVID-19 transmission prevention behaviors and perceptions over time. We observed higher levels of stress, anxiety, fatigue, and feelings of being unsafe at all time points relative to pre-pandemic, mirroring trends for social isolation-related outcomes. Further, compared to the pre-pandemic period, lower levels of physical activity and perceived productivity and higher levels of non-work screen time and alcohol consumption were observed. Employees mostly reported believing COVID-19 prevention behaviors are important, and most reported being vaccinated. A unique aspect of our study is the timing of each COVID-19 surge relative to each CAPTURE Survey deployment time point. Our baseline and 12-mo surveys were deployed in late 2020/early 2021 and late 2021/early 2022, respectively, in the middle of COVID-19 surges, while our 3-mo and 6-mo surveys were deployed in mid-2021 when vaccinations were becoming widespread and/or COVID-19 cases were far lower.

Although psychosocial outcomes were poorer at all time points relative to pre-pandemic, the observations during the pandemic were more nuanced. Specifically, the highest levels of stress and anxiety were reported at baseline and 12 months, with lower levels at three and six months. This mirrors the aforementioned COVID-19 surges that were present during our baseline and 12-mo surveys. Similarly, Kujawa et al. [33] noted that depression and anxiety levels decreased among U.S. adults between May 2020 and June 2020, mirroring trends in another study assessing these outcomes from May 2020 to August 2020 [34]. Somewhat contrastingly, a larger one-year longitudinal study from May 2020 to April 2021 observed higher levels of depression and anxiety for most of the survey period but noted some evidence of slightly lower levels near the end of the study, around the same time as our 3-mo survey [35]. Surges in COVID-19 forced many individuals to isolate. The changes that we and others have observed across time in psychosocial outcomes may be associated with isolation. Indeed, our observations not only noted that employees felt isolated and a lack of companionship, but also that nearly half could have used more emotional support than received. During the summer months of 2021, coinciding with our 3- and 6-mo survey deployments, more opportunities to safely gather outdoors may have reduced these feelings of isolation and contributed to the slightly better psychosocial health we observed at these time points. It may also be that individuals were less fearful of being exposed to the virus at these time points, further bolstered by our COVID-19-related perception results. Continued investigation is needed.

In the current study, we observed reported engagement in the recommended amounts of several health behaviors to be lower at all time points relative to the pre-pandemic period. Similar to our physical activity observations, other studies have reported a decline in this health behavior during the pandemic [9, 36]. Higher physical activity participation has been shown to be associated with fewer mental health problems during the COVID-19 pandemic [37], possibly suggesting that lower participation in physical activity may have contributed to some of our observations of poorer psychosocial health. Other studies have noted increased sleep duration from pre-pandemic to during the pandemic [38]. We observed a similar trend for sleep duration from pre-pandemic to baseline. This may have been attributable to less commute time to work and thus more time available to sleep. However, in the follow-up time points, this observation did not hold, as sleep duration in our sample returned to the lower durations observed pre-pandemic. This could perhaps be due to a shift back to working in the office periodically.

Non-work screen time and alcohol consumption both trended upward relative to pre-pandemic and remained higher. Wagner et al. [39] reported an average increase in non-work screen time of 2.6 h/week from pre-pandemic to during the pandemic among adults, while Chen et al. [9] noted that the percentage of adults engaged in ≥ 4 h/day increased by 20% over this time period. In our study, we observed the prevalence of employees engaging in > 2 h of non-work screen time to be 20% higher than pre-pandemic by our 12-month time point. It is unclear if this increased non-work screen time is related to less time spent commuting during the pandemic or more subtle reasons, such as an increase in social media app usage to reduce feelings of isolation or the greater use of television watching as a coping mechanism. Research has noted increased screentime during the COVID-19 pandemic [40], with other research noting the detrimental impact of screentime on psychosocial health [41, 42]. As our sample reported either hybrid work or being nearly entirely WFH, a plausible connection might also exist between our screentime observations and psychosocial health. Finally, compared to pre-pandemic, we observed an increase of ~ 4–7% at all time points for those consuming ≥ 7 alcoholic drinks/week compared to pre-pandemic. This is a similar increase as reported by Chen et al. [9]. Notably, Barbosa et al. [43] conducted a similar cross-sectional survey before and after the enactment of stay-at-home orders during the COVID-19 pandemic and found higher rates of binge drinking among U.S adults. Further research is recommended to continue to monitor this trend and consider interventions or policy changes.

While our results regarding the COVID-19-related prevention behaviors were encouraging, with remarkably high engagement in the most common behaviors (e.g., masking, physically distancing, getting vaccinated), the observed poorer psychosocial outcomes and health behavior engagement are notable. Employed individuals spend most of their waking hours working. Thus, companies would do well to invest in mental and/or physical health programs to improve the health of their employees. This is particularly salient given the widespread mental health difficulties caused by the pandemic and the fact that engaging in poorer health behaviors can contribute to worsened cardiometabolic health. Indeed, significantly higher blood pressure levels have been reported for U.S. adults during the pandemic [44, 45].

This study has a few limitations deserving consideration. First, while the surveys were only available to each company’s employees for a three-week period, we deployed the survey to the eight companies across an approximate three-month timespan at each time point. Given the transient nature of COVID-19 surges, responses may have been biased due to when a given company’s employees completed a given time point’s survey. Second, we surveyed a convenience sample of companies, with > 50% of employees having master’s, professional, or doctoral degrees, limiting generalizability to populations of lower socioeconomic status. Third, less than half of the initial employee sample responded by the final deployment of the CAPTURE Survey at the 12-month time point (1,687 at 12 months vs. 3,607 at baseline). This raises the possibility of overestimation, as employees having greater psychosocial and behavioral difficulties due to the COVID-19 pandemic might be more likely to complete the follow-up surveys. Finally, > 90% of the employees were fully vaccinated by the 12-mo survey. Survey responses among samples who are largely unvaccinated may differ. Our study has strengths in the: (1) longitudinal design; (2) large sample size; and (3) high response rate relative to other surveys of this type. Importantly, our study adds to the scant literature on how COVID-19 has impacted the psychosocial and behavioral responses in a sample of largely non-healthcare employees over time, an assessment we do not believe has been completed to date.

## Conclusions

The COVID-19 pandemic appears to have had a harmful impact on psychosocial and behavioral health across employees in the non-healthcare workforce. Specifically, our results suggest that individuals have experienced little psychological respite and fewer opportunities for proper health behavior engagement despite our sample’s remarkably high vaccination and socioeconomic statuses. Companies thus need to prioritize programs targeted at employee health before harmful long-term effects on employees’ health manifest.

## Electronic supplementary material

Below is the link to the electronic supplementary material.

Supplementary Material 1

## Data Availability

Our data are available upon specific request to the corresponding author.

## Abbreviations

CAPTURE: Characterizing Awareness of SARS-CoV-2 PrevenTion and Understanding Responses and Experiences (CAPTURE) Survey
COVID-19: coronavirus disease-2019
WFH: work from home
