# Supplementary material for: Psychosocial and behavioral outcomes in the adult workforce during the COVID-19 pandemic: a 1-Year longitudinal survey
Source: BMC Public Health. 2023 Apr 3;23:634. doi: 10.1186/s12889-023-15536-8 (PMC10068713; doi:10.1186/s12889-023-15536-8)
Supplement: Supplementary file 1 — Supplementary Material 1 [file 12889_2023_15536_MOESM1_ESM.pdf]

Supplementary Material Portion #1:

STROBE Statement—Checklist of items that should be included in reports of ***cohort studies***

	Item No	Recommendation	Page No
**Title and abstract**	1	(*a*) Indicate the study's design with a commonly used term in the title or the abstract<br>(*b*) Provide in the abstract an informative and balanced summary of what was done and what was found	1-3
**Introduction**			
Background/rationale	2	Explain the scientific background and rationale for the investigation being reported	4-5
Objectives	3	State specific objectives, including any prespecified hypotheses	5
**Methods**			
Study design	4	Present key elements of study design early in the paper	6-7
Setting	5	Describe the setting, locations, and relevant dates, including periods of recruitment, exposure, follow-up, and data collection	6-7
Participants	6	(*a*) Give the eligibility criteria, and the sources and methods of selection of participants. Describe methods of follow-up<br>(*b*) For matched studies, give matching criteria and number of exposed and unexposed	7
Variables	7	Clearly define all outcomes, exposures, predictors, potential confounders, and effect modifiers. Give diagnostic criteria, if applicable	7-9; Citation #5
Data sources/ measurement	8*	For each variable of interest, give sources of data and details of methods of assessment (measurement). Describe comparability of assessment methods if there is more than one group	7-9; Citation #5
Bias	9	Describe any efforts to address potential sources of bias	9-10
Study size	10	Explain how the study size was arrived at	9-10
Quantitative variables	11	Explain how quantitative variables were handled in the analyses. If applicable, describe which groupings were chosen and why	9-10
Statistical methods	12	(*a*) Describe all statistical methods, including those used to control for confounding<br>(*b*) Describe any methods used to examine subgroups and interactions<br>(*c*) Explain how missing data were addressed<br>(*d*) If applicable, explain how loss to follow-up was addressed<br>(*e*) Describe any sensitivity analyses	9-10
**Results**			
Participants	13*	(a) Report numbers of individuals at each stage of study—eg numbers potentially eligible, examined for eligibility, confirmed eligible, included in the study, completing follow-up, and analysed<br>(b) Give reasons for non-participation at each stage<br>(c) Consider use of a flow diagram	10
Descriptive data	14*	(a) Give characteristics of study participants (eg demographic, clinical, social) and information on exposures and potential confounders<br>(b) Indicate number of participants with missing data for each variable of interest<br>(c) Summarise follow-up time (eg, average and total amount)	10-13; Supplementary Material

Outcome data	15*	Report numbers of outcome events or summary measures over time	11-13; Supplementary Material

Main results	16	(*a*) Give unadjusted estimates and, if applicable, confounder-adjusted estimates and their precision (eg, 95% confidence interval). Make clear which confounders were adjusted for and why they were included<br>(*b*) Report category boundaries when continuous variables were categorized<br>(*c*) If relevant, consider translating estimates of relative risk into absolute risk for a meaningful time period	11-13; Supplementary Material
Other analyses	17	Report other analyses done—eg analyses of subgroups and interactions, and sensitivity analyses	11-13; Supplementary Material
**Discussion**			
Key results	18	Summarise key results with reference to study objectives	13-14
Limitations	19	Discuss limitations of the study, taking into account sources of potential bias or imprecision. Discuss both direction and magnitude of any potential bias	16-17
Interpretation	20	Give a cautious overall interpretation of results considering objectives, limitations, multiplicity of analyses, results from similar studies, and other relevant evidence	14-16
Generalisability	21	Discuss the generalisability (external validity) of the study results	14-16
**Other information**			
Funding	22	Give the source of funding and the role of the funders for the present study and, if applicable, for the original study on which the present article is based	19

*Give information separately for exposed and unexposed groups.

Note: An Explanation and Elaboration article discusses each checklist item and gives methodological background and published examples of transparent reporting. The STROBE checklist is best used in conjunction with this article (freely available on the Web sites of PLoS Medicine at http://www.plosmedicine.org/, Annals of Internal Medicine at http://www.annals.org/, and Epidemiology at http://www.epidem.com/). Information on the STROBE Initiative is available at http://www.strobe-statement.org.

Supplementary Material Portion #2: COVID20 Aim 3 Survey 12-month

Start of Block: Consent

Research Study: Working During the COVID-19 Pandemic: Characterizing Awareness of SARS CoV-2 PrevenTion and Understanding Responses and Experiences (CAPTURE) Survey

Please click the link to read the Consent Form and sign below.

I have read the Consent Form and agree to participate in this research study.

Q1 Are you aged 18 years or older?

- ○ Yes (1)
- ○ No (2)

Skip To: End of Survey If Q1 = 2

Email Please enter the email address* at which you received this survey:

**Please note that we will only ask for your email so that we may contact you with an invitation to complete the survey at the final 12-month timepoint. All emails are maintained in a secure, password-protected database that will only be used to contact you at the future timepoints. Emails will be deidentified (stripped) from all responses prior to data analysis. Please see consent form for more information.*

____

Q2 Please state the name of the company you work for.

____

Q3 In which state do you currently work in?

▼ Alabama (1) ... I do not reside in the United States (53)

Skip To: End of Survey If Q3 = 53

End of Block: Consent

Start of Block: Inclusion/Exclusion

Working During the COVID-19 Pandemic: The CAPTURE Survey

Characterizing Awareness of SARS-CoV-2 PrevenTion and Understanding Responses and Experiences

Thank you for your willingness to complete this survey! It should take no longer than 15 minutes to complete. As you will see, some questions are personal in nature, and others assess work-related behaviors as they relate to the current coronavirus disease-19 (**COVID-19**) pandemic. Severe acute respiratory syndrome coronavirus 2 (**SARS-CoV-2**), the virus causing COVID-19, refers to the illness caused by the novel coronavirus that was first identified in 2019.

We realize there are many different opinions on both the severity of, and responses to, the pandemic. Please know that **your answers to these questions are strictly confidential** and study results will only be presented in summary form. With that, we ask you to be honest in your responses.

When answering these questions, please only think about your work at ${Q2/ChoiceTextEntryValue}.

We very much appreciate your time and honesty.

If you have any questions while taking this study, please email the study coordinator.

Study contacts:

Well Living Lab: Araliya Senerat at senerat.araliya@mayo.edu.
University of Minnesota: Sarah Rydell at rydel004@umn.edu.

Q4 Are you an employee of ${Q2/ChoiceTextEntryValue} and currently employed?

- ◯ Yes (1)
- ◯ No (2)

Skip To: End of Survey If Q4 = 2

---

indoors On average, what percentage (%) of time do you spend indoors* working in your current role at ${Q2/ChoiceTextEntryValue}? **indoors refers to working inside a building or home and not outside. The percentage is out of your total work hours.*

	0 10 20 30 40 50 60 70 80 90 100
% of time indoors ()	

Skip To: End of Survey If indoors [3] < 50

End of Block: Inclusion/Exclusion

Start of Block: Description of work environment

Q5 How many hours per week, on average, do you work at this job?

- ○ 0 hours/wk (1)
- ○ 1-8 hours (2)
- ○ 9-16 (3)
- ○ 17-24 (4)
- ○ 25-32 (5)
- ○ 33-40 (6)
- ○ 41-60 (7)
- ○ 61 or more (8)
- ○ Prefer not to answer (99)

Q6
On average, how many hours per week do you currently have face-to-face, in-person interactions with either your coworkers or the public while completing your job-related duties?

- ○ 0 hours/wk (1)
- ○ 1-8 hours (2)
- ○ 9-16 (3)
- ○ 17-24 (4)
- ○ 25-32 (5)
- ○ 33-40 (6)
- ○ 41-60 (7)
- ○ 61 or more (8)
- ○ Prefer not to say (99)

Q7 Please choose the category that best describes your <u>main</u> job. If none of the categories fits you exactly, please respond with the closest category to your experience. (Select only <u>one</u>.)

- ◯ Executive, administrator, or senior manager (e.g., CEO, sales, VP, plant manager) (1)
- ◯ Professional (e.g., engineer, accountant, systems analyst) (2)
- ◯ Technical support (e.g., lab technician, legal assistant, computer programmer) (3)
- ◯ Sales (e.g., sales representative, stockbroker, retail sales) (4)
- ◯ Clerical and administrative support (e.g., secretary, billing clerk, office supervisor) (5)
- ◯ Service occupation (e.g., security officer, carpenter, machinist) (6)
- ◯ Chemical/Production Operator (e.g., shift supervisors and hourly employees) (7)
- ◯ Laborer (e.g., truck driver, construction worker) (8)
- ◯ Food industry service occupation (e.g. server, chef, cook) (9)
- ◯ Other (10) ____
- ◯ Prefer not to answer (99)

Q8 **<u>At the time the second CAPTURE Survey was sent 3 months ago</u>**, what percentage of your work hours (weekly average) were you working at home?

Prefer not to answer

	0 10 20 30 40 50 60 70 80 90 100
Percentage (%) at home ()	

Q9 **<u>Currently at this time</u>**, what percentage of your work hours (weekly average) are you working at home?

Prefer not to answer

	0 10 20 30 40 50 60 70 80 90 100
Percentage (%) at home ()	

Q10 At ${Q2/ChoiceTextEntryValue}, are you now:

- working full time, same hours as 3 months ago (1)
- working full time, different hours than 3 months ago (2)
- working part-time, same hours as before 3 months ago (3)
- working part-time, different hours than 3 months ago (4)
- retired (5)
- unemployed (6)
- permanently laid off (7)
- furloughed/furloughed previously (or temporarily laid off) (8)

Q11 Please indicate whether any of these situations apply to you (check all that apply):

- ▢ Working remotely or from home (1)
- ▢ Working back at the office (2)
- ▢ Working a combination model (part-time at home & part-time at the office) (3)
- ▢ Had to get a second job (4)
- ▢ Working a second job, same hours as 3 months ago (5)
- ▢ Working a second job, different hours than 3 months ago (6)
- ▢ At increased risk of getting COVID-19 in your job (7)

X→

Q12 Please indicate the extent to which you view the COVID-19 outbreak as having either a positive or negative impact on your work.

- ○ Extremely negative (1)
- ○ Moderately negative (2)
- ○ Somewhat negative (3)
- ○ No impact (4)
- ○ Slightly positive (5)
- ○ Moderately positive (6)
- ○ Extremely positive (7)
- ○ Prefer not to answer (99)

End of Block: Description of work environment

Start of Block: Socioeconomic impact of COVID questionnaire_060120

As a direct result of the COVID pandemic:

Display This Question:

If Q10 = 4

X→

Q13 You said you were working part time at ${Q2/ChoiceTextEntryValue}, but different hours 3 months ago. Are you working more hours or fewer hours?

- More hours (1)
- Fewer hours (2)
- Prefer not to answer (99)

Display This Question:

If Q11 = 6

X→

Q14 You said you were working part time, but different hours at your second job 3 months ago. Are you working more hours or fewer hours?

- More hours (1)
- Fewer hours (2)
- Prefer not to answer (99)

X→

Q15 Have you lost health insurance or other coverage for medical care?

- Yes (1)
- No (2)
- Don't Know (3)
- Prefer not to answer (99)

End of Block: Socioeconomic impact of COVID questionnaire_060120

Start of Block: Personal Covid prevention behaviors

X→

Q16
In the past month, I have taken the following precautions **at my company's workplace**:

	Never (1)	Rarely (2)	Sometimes (3)	Often (4)	Always (5)	Not Applicable (99)
Worn a mask of any type (Q16_1)	○	○	○	○	○	○
Worn gloves (Q16_2)	○	○	○	○	○	○
Washed my hands regularly (Q16_3)	○	○	○	○	○	○
Physically distanced from coworkers or public (Q16_4)	○	○	○	○	○	○
Disinfected surfaces at which I primarily work (Q16_5)	○	○	○	○	○	○
Monitored symptoms prior to work (e.g., measuring temperature) (Q16_6)	○	○	○	○	○	○
Other, please describe: (Q16_7)	○	○	○	○	○	○

Q17
In the past month, how often have you observed your co-workers taking the following precautions **at your company's workplace**?

	Never (1)	Rarely (2)	Sometimes (3)	Often (4)	Always (5)	Not Applicable (99)
Worn a mask of any type (Q17_1)	○	○	○	○	○	○
Worn gloves (Q17_2)	○	○	○	○	○	○
Washed their hands regularly (Q17_3)	○	○	○	○	○	○
Physically distanced from coworkers or public (Q17_4)	○	○	○	○	○	○
Disinfected surfaces at which they primarily work (Q17_5)	○	○	○	○	○	○
Other, please describe: (Q17_6)	○	○	○	○	○	○

End of Block: Personal Covid prevention behaviors

Start of Block: Workplace culture, practices, training, PPE provided, etc

X→

Q18
Within the past 1 month, ${Q2/ChoiceTextEntryValue} has **provided**:

	Never (1)	Rarely (2)	Sometimes (3)	Often (4)	Always (5)	Not Applicable (99)
N95 masks (Q18_1)	o	o	o	o	o	o
Surgical masks (Q18_2)	o	o	o	o	o	o
Cloth masks (Q18_3)	o	o	o	o	o	o
Gloves (Q18_4)	o	o	o	o	o	o
Hand sanitizer (Q18_5)	o	o	o	o	o	o
Hand washing instructions (Q18_6)	o	o	o	o	o	o
Physical distancing instructions (Q18_7)	o	o	o	o	o	o
Cleaning/disinfecting products for surfaces (Q18_8)	o	o	o	o	o	o
Tools to monitor symptoms prior to work (e.g., thermometer to measure temperature) (Q18_9)	o	o	o	o	o	o
Other, please describe: (Q18_10)	o	o	o	o	o	o

Q19

Within the past 1 month, ${Q2/ChoiceTextEntryValue} has **promoted**:

	Never (1)	Rarely (2)	Sometimes (3)	Often (4)	Always (5)	Not Applicable (99)
N95 masks (Q19_1)	○	○	○	○	○	○
Surgical masks (Q19_2)	○	○	○	○	○	○
Cloth masks (Q19_3)	○	○	○	○	○	○
Gloves (Q19_4)	○	○	○	○	○	○
Hand sanitizer (Q19_5)	○	○	○	○	○	○
Hand washing (Q19_6)	○	○	○	○	○	○
Physical distancing (Q19_7)	○	○	○	○	○	○
Surface cleaning/disinfecting (Q19_8)	○	○	○	○	○	○
Monitoring of symptoms prior to work (e.g., thermometer to measure temperature) (Q19_9)	○	○	○	○	○	○
Encouraging staff to get vaccinated (Q19_10)	○	○	○	○	○	○
Sponsoring vaccination efforts (Q19_11)	○	○	○	○	○	○
Other, please describe: (Q19_12)	○	○	○	○	○	○

Q20 What best describes the COVID-19 prevention training that your employer has provided **within the last 3 months**?

- ☐ ⊗None (1)
- ☐ Web training (2)
- ☐ In-person training (3)
- ☐ Reading materials (4)
- ☐ Other, please describe (5) ____
- ☐ Prefer not to answer (99)

End of Block: Workplace culture, practices, training, PPE provided, etc

Start of Block: Perceptions of importance/efficacy of behaviors & practices

Q21 How important do you think the following practices are in the prevention of the spread of COVID-19?:

	Not important (1)	Somewhat important (2)	Moderately important (3)	Very important (4)	Prefer not to answer (99)
Wearing a mask (Q21_1)	○	○	○	○	○
Wearing gloves (Q21_2)	○	○	○	○	○
Handwashing (Q21_3)	○	○	○	○	○
Physical distancing (Q21_4)	○	○	○	○	○
Disinfecting surfaces (Q21_5)	○	○	○	○	○
Getting the COVID-19 vaccine (Q21_6)	○	○	○	○	○
Other, please describe: (Q21_7)	○	○	○	○	○

End of Block: Perceptions of importance/efficacy of behaviors & practices

Start of Block: Psychosocial experiences - Before & During

Q22 How much did you experience the following feelings due to your work-related duties **<u>over the past month?</u>**

	Never (1)	Rarely (2)	Moderately (3)	Quite a Bit (4)	All the time (5)	Prefer not to answer (99)
Stress (Q22_1)	○	○	○	○	○	○
Anxiety (Q22_2)	○	○	○	○	○	○
Fatigue (Q22_3)	○	○	○	○	○	○
Feeling unsafe (Q22_4)	○	○	○	○	○	○

Q23 How much do you agree or disagree with the following statements?

	Strongly Disagree (1)	Disagree (2)	Neutral (3)	Agree (4)	Strongly Agree (5)
Thinking about the coronavirus (COVID-19) makes me feel threatened (Q23_1)	o	o	o	o	o
I am afraid of the coronavirus (COVID-19) (Q23_2)	o	o	o	o	o
I am stressed around other people because I worry I'll catch the coronavirus (COVID-19) (Q23_3)	o	o	o	o	o
Getting the COVID-19 vaccine is very important to me (Q23_4)	o	o	o	o	o

Q24 How would you describe your level of **productivity** in your job **over the past month?**

- Low (1)
- Slightly below average (2)
- Average (3)
- Slightly above average (4)
- High (5)
- Prefer not to answer (99)

Q25 How often have you felt the following **at work over the past month?**

	Hardly ever (1)	Some of the time (2)	Often (3)	Prefer not to answer (99)
A lack of companionship (Q25_1)	○	○	○	○
A feeling of being left out (Q25_2)	○	○	○	○
A feeling of being isolated from others (Q25_3)	○	○	○	○

Q26
How many **minutes** per day did you spend physically active (i.e., walking, jogging, swimming, gardening, house-chores) **over the past month**?

- 0-30 minutes per day (1)
- 30-60 minutes per day (2)
- 60-90 minutes per day (3)
- 90-120 minutes per day (4)
- >120 minutes per day (5)
- Prefer not to answer (99)

Q27
How many **hours** per day did you watch television, use the computer for non-work, utilize your phone for entertainment, play video games **over the past month?**

- 0-1 hour per day (1)
- 1-2 hours per day (2)
- 2-4 hours per day (3)
- >5 hours per day (4)
- Prefer not to answer (99)

Q28
On average, how many **hours** per night of sleep did you get **<u>over the past month?</u>**

- <6 hours per night (1)
- 7 hours per night (2)
- 8 hours per night (3)
- 9 hours per night (4)
- >10 hours per night (5)
- Prefer not to answer (99)

Q29

How often did you have any kind of drink containing alcohol **on average each week <u>over the past month</u>**?

By a drink we mean half an ounce of absolute alcohol (e.g. a 12 ounce can or glass of beer or cooler, a 5 ounce glass of wine, or a drink containing 1 shot of liquor).

Choose only one.

- ○ 0 drinks/week (1)
- ○ 1-3 drinks/week (2)
- ○ 4-6 drinks/week (3)
- ○ 7-9 drinks/week (4)
- ○ 10-12 drinks/week (5)
- ○ 13-15 drinks/week (6)
- ○ 16-18 drinks/week (7)
- ○ 19-21 drinks/week (8)
- ○ >21 drinks/week (9)
- ○ Prefer not to answer (99)

Q30
How would you categorize your use of the following tobacco/nicotine products **over the past month**?

	Increased (1)	Decreased (2)	Stayed the same (3)	Never used (4)	Prefer not to answer (99)
Cigarettes (Q30_1)	○	○	○	○	○
E-cigarettes (Q30_2)	○	○	○	○	○
Pipe, cigars, or cigarillos (Q30_3)	○	○	○	○	○
Smokeless tobacco (e.g. snuff, chewing tobacco, dip) (Q30_4)	○	○	○	○	○

X→

Q31 How worried are you that you'll contract COVID-19 while at work?

○ Not at all worried (1)

○ Somewhat worried (2)

○ Moderately worried (3)

○ Very worried (4)

○ Prefer not to answer (99)

○ I have had COVID-19 likely due to workplace exposure (5)

X→

Q32 How worried are you that you could be an asymptomatic carrier of COVID-19 and may be spreading it to other people **at your company's workplace**?

- ◯ Not at all worried (1)
- ◯ Somewhat worried (2)
- ◯ Moderately worried (3)
- ◯ Very worried (4)
- ◯ Prefer not to answer (99)

Support_1 Can you count on anyone to provide you with emotional support, such as talking over problems or helping you make a difficult decision?

- ◯ Yes (1)
- ◯ No (2)
- ◯ I don't need help (3)

Display This Question:

If Support_1 = 1

Support_2 In the last 12 months, could you have used more emotional support than you received?

- ◯ Yes (1)
- ◯ No (2)

Display This Question:

If Support_2 = 1

Support_3 Would you say that you could have used ________ more social support?

- ◯ A little (1)
- ◯ Some (2)
- ◯ A lot (3)

End of Block: Psychosocial experiences - Before & During

Start of Block: CARDIA/ECHO

X→

Vaccine1 Have you received a COVID-19 vaccine?

- ◯ Yes, one dose (1)
- ◯ Yes, two doses (2)
- ◯ No (3)
- ◯ Prefer not to answer (99)

Display This Question:

If Vaccine1 = 1

Or Vaccine1 = 2

X→

Vaccine2 Which COVID-19 vaccine did you receive for the **first or only dose**?

- ◯ Pfizer-BioNTech (1)
- ◯ Moderna (2)
- ◯ Johnson & Johnson (3)
- ◯ Other, please state the name: (4) ____
- ◯ I don't know/Prefer not to answer (99)

Display This Question:

If Vaccine2 = 3

Or Vaccine2 = 2

Or Vaccine2 = 1

JS X→

Vaccine3 What was the date of your **first or only** COVID-19 vaccine dose? (an estimated date is fine)

	Month	Day	Year
1st Dose (1)	▼ January (1 ... December (12)	▼ 1 (1 ... 31 (31)	▼ 2020 (1 ... 2022 (3)

Display This Question:

If Vaccine1 = 2

X→

Vaccine4 Which COVID-19 vaccine did you receive for the **second dose**?

○ Pfizer-BioNTech (1)

○ Moderna (2)

○ Johnson & Johnson (3)

○ Other, please state the name: (4)

____

○ I don't know/Prefer not to answer (99)

Display This Question:

If Vaccine1 = 2

JS

Vaccine5 What was the date of your **second** COVID-19 vaccine dose? (an estimated date is fine)

	Month	Day	Year
2nd Dose (1)	▼ January (1 ... December (12)	▼ 1 (1 ... 31 (31)	▼ 2020 (1 ... (4)

Display This Question:

If Vaccine1 = 1

Or Vaccine1 = 2

Vaccine6 Did you receive a COVID-19 vaccine booster shot?

◯ Yes (1)

◯ No (2)

◯ Prefer not to answer (3)

Display This Question:

If Vaccine6 = 1

X→

Vaccine7 Which type of COVID-19 vaccine did you receive for your **booster?**

◯ Pfizer-BioNTech (1)

◯ Moderna (2)

◯ Johnson & Johnson (3)

◯ Other, please state the name: (4) ____

◯ I don't know/Prefer not to answer (99)

Display This Question:

If Vaccine6 = 1

JS

Vaccine6 What was the date of your **booster** COVID-19 vaccine dose? (an estimated date is fine)

	Month	Day	Year
2nd Dose (1)	▼ January (1 ... December (12)	▼ 1 (1 ... 31 (31)	▼ 2020 (1 ... (4)

To help us better understand the health of all study participants during the COVID-19 pandemic, we would like to ask you questions about your possible exposure to this new virus. We use the term COVID-19 to refer to the illness caused by the novel coronavirus that was first identified in 2019. This virus is also called SARS-CoV-2.

Below is a list of symptoms that may be related to COVID-19. Some of these may also occur with other conditions such as allergies, colds and flu or when taking certain medications.

X→

Q33 Have you had any of these symptoms for longer than several hours or more than is usual for you, **within the last 3 months?**

	No (1)	Yes (2)	Prefer not to answer (99)
Fever (≥100.4°F) (Q33_1)	○	○	○
Persistent cough (Q33_2)	○	○	○
Unusual shortness of breath or difficulty breathing (Q33_3)	○	○	○
Chills or sweats (Q33_4)	○	○	○
Headache (Q33_5)	○	○	○
Sore throat (Q33_6)	○	○	○
Unusually hoarse (Q33_7)	○	○	○
Loss of smell (Q33_8)	○	○	○
Loss of taste (Q33_9)	○	○	○
Chest pain/tightness (Q33_10)	○	○	○
Muscle aches (Q33_11)	○	○	○
Abdominal pain (Q33_12)	○	○	○
Diarrhea (Q33_13)	○	○	○
Confusion (Q33_14)	○	○	○
Malaise or general feeling of illness, discomfort or	○	○	○

uneasiness (Q33_15)			
Unusual fatigue (Q33_16)	○	○	○

Carry Forward Selected Choices from "Q33"

X→

Q34 If **YES**, how severe was this symptom?

	Mild (1)	Moderate (2)	Severe (3)	Prefer not to answer (99)
Fever (≥100.4°F) (Q34_x1)	○	○	○	○
Persistent cough (Q34_x2)	○	○	○	○
Unusual shortness of breath or difficulty breathing (Q34_x3)	○	○	○	○
Chills or sweats (Q34_x4)	○	○	○	○
Headache (Q34_x5)	○	○	○	○
Sore throat (Q34_x6)	○	○	○	○
Unusually hoarse (Q34_x7)	○	○	○	○
Loss of smell (Q34_x8)	○	○	○	○
Loss of taste (Q34_x9)	○	○	○	○
Chest pain/tightness (Q34_x10)	○	○	○	○
Muscle aches (Q34_x11)	○	○	○	○
Abdominal pain (Q34_x12)	○	○	○	○
Diarrhea (Q34_x13)	○	○	○	○
Confusion (Q34_x14)	○	○	○	○

Malaise or general feeling of illness, discomfort or uneasiness (Q34_x15)	○	○	○	○
Unusual fatigue (Q34_x16)	○	○	○	○

Q35 Which of the following statements apply to you within the **past 3 months** (check all that apply)?

- ☐ I do not think I had COVID-19 (1)
- ☐ I tested negative for COVID-19 (2)
- ☐ I tested positive for COVID-19 (3)
- ☐ I suspected that I had COVID-19 but I was never tested (4)
- ☐ I called my health care provider because I thought I might have COVID-19 and I was told to stay home (quarantine) (5)
- ☐ I went to a clinic, emergency room, or hospital because I had COVID-19 symptoms (6)
- ☐ Prefer not to answer (99)

Q36 Have you ever had a COVID-19 test?

- Yes (1)
- No (2)
- Prefer not to answer (99)

Display This Question:

If Q36 = 1

Q36a Please select the following COVID-19 tests you have received (select all that apply):

- Nasal swab test (1)
- Spit test (2)
- Rapid test (3)
- Blood test (also known as an antibody test) (4)
- Other, please describe (5) ____

Display This Question:

If Q36 = 1

Q36b Do you get routinely tested for COVID-19 **for work?**

- Yes (1)
- No (2)
- Prefer not to answer (99)

Display This Question:

If Q36b = 1

Q36c **At what frequency** do you get tested for COVID-19 at work?

- ○ Once per week or more (1)
- ○ Once every two weeks (2)
- ○ Once per month (3)
- ○ Other, please describe (4) ____

Q38 In what ways has the COVID-19 outbreak affected your overall healthcare (e.g., primary care provider, dentist, eye doctor, therapist, etc.) **over the past 3 months**? ***(Mark all that apply)***

- ▢ I did not go to **some** healthcare appointments because I was concerned about entering my healthcare providers' office (1)
- ▢ I did not go to **any** healthcare appointments because I was concerned about entering my healthcare providers' office (2)
- ▢ My healthcare provider(s) continued phone or online visits (3)
- ▢ My healthcare provider(s) changed to phone or online visits (4)
- ▢ My healthcare provider(s) told me to self-isolate or quarantine (5)
- ▢ ⊗None of these apply (6)
- ▢ ⊗Prefer not to answer (99)

Q39 Which of the following behaviors have you **done** because of the COVID-19 outbreak **within the last 3 months**? ***(Mark all that apply)***

- ▢ Less in-person contact with people inside my home (that is, you are quarantined separately from one or more family or household members) (1)
- ▢ Less in-person contact with family who live outside the home (2)
- ▢ Less in-person contact with friends (3)
- ▢ Less in-person contact with colleagues at work (4)
- ▢ Less in-person events in the community, including religious events (5)
- ▢ ⊗None of these apply (6)
- ▢ ⊗Prefer not to answer (99)

End of Block: CARDIA/ECHO

Start of Block: Health Related Questions

X→

Q40 In general, would you say your health is:

- ○ Poor (1)
- ○ Fair (2)
- ○ Good (3)
- ○ Very Good (4)
- ○ Excellent (5)
- ○ Prefer not to answer (99)

X→

Q41 Have you ever been told by a doctor that you have...

	Yes (1)	No (2)	Not Sure (3)	Prefer not to answer (99)
Heart disease or angina (Q41_1)	○	○	○	○
High blood pressure (hypertension) (Q41_2)	○	○	○	○
Stroke (Q41_3)	○	○	○	○
Diabetes (high blood sugar) (Q41_4)	○	○	○	○
Cancer (Q41_5)	○	○	○	○
High cholesterol (Q41_6)	○	○	○	○
An anxiety disorder (e.g. GAD) (Q41_7)	○	○	○	○
A mood disorder (e.g. depression) (Q41_8)	○	○	○	○

X→

Q42 What is your gender?

○ Woman (1)

○ Man (2)

○ Other/Non-binary (3)

○ Prefer not to answer (99)

End of Block: Health Related Questions

Start of Block: Demographic Block

Demographic Information

Q43 What is your current age?

____

Q44 Are you Hispanic or Latino/Latina?

- Yes (1)
- No (2)
- I prefer not to answer (99)

Q45 Which of the following best describes you? (Check all that apply)

- ▢ Asian or Asian-American (1)
- ▢ Black or African-American (2)
- ▢ Hawaiian or Pacific Islander (3)
- ▢ Native American or Alaskan Native (4)
- ▢ Hispanic or Latino/Latina (5)
- ▢ White (6)
- ▢ Other (please specify) (7) ____
- ▢ I prefer not to answer (99)

Q46 What is your current marital status?

- ○ Single (1)
- ○ Married or partnered (2)
- ○ I prefer not to answer (99)

Q47 What is the highest level of schooling you completed?

- ○ Less than High School (1)
- ○ Obtained GED (2)
- ○ High School Graduate (diploma) (3)
- ○ Completed some college credit, but no degree (4)
- ○ Associate degree (5)
- ○ Bachelor's degree (6)
- ○ Master's, Professional, or Doctoral degree (7)
- ○ I prefer not to answer (99)

End of Block: Demographic Block

Start of Block: Open ended questions

Q48 Do you have any other thoughts or feelings regarding working during the COVID-19 pandemic that you do not feel might have been captured within this survey? If so, please describe.

____

____

____

____

____

End of Block: Open ended questions

Supplementary Material Portion #3

Supplemental Figure 1. Feelings at Work for Full Dataset Across Timepoints

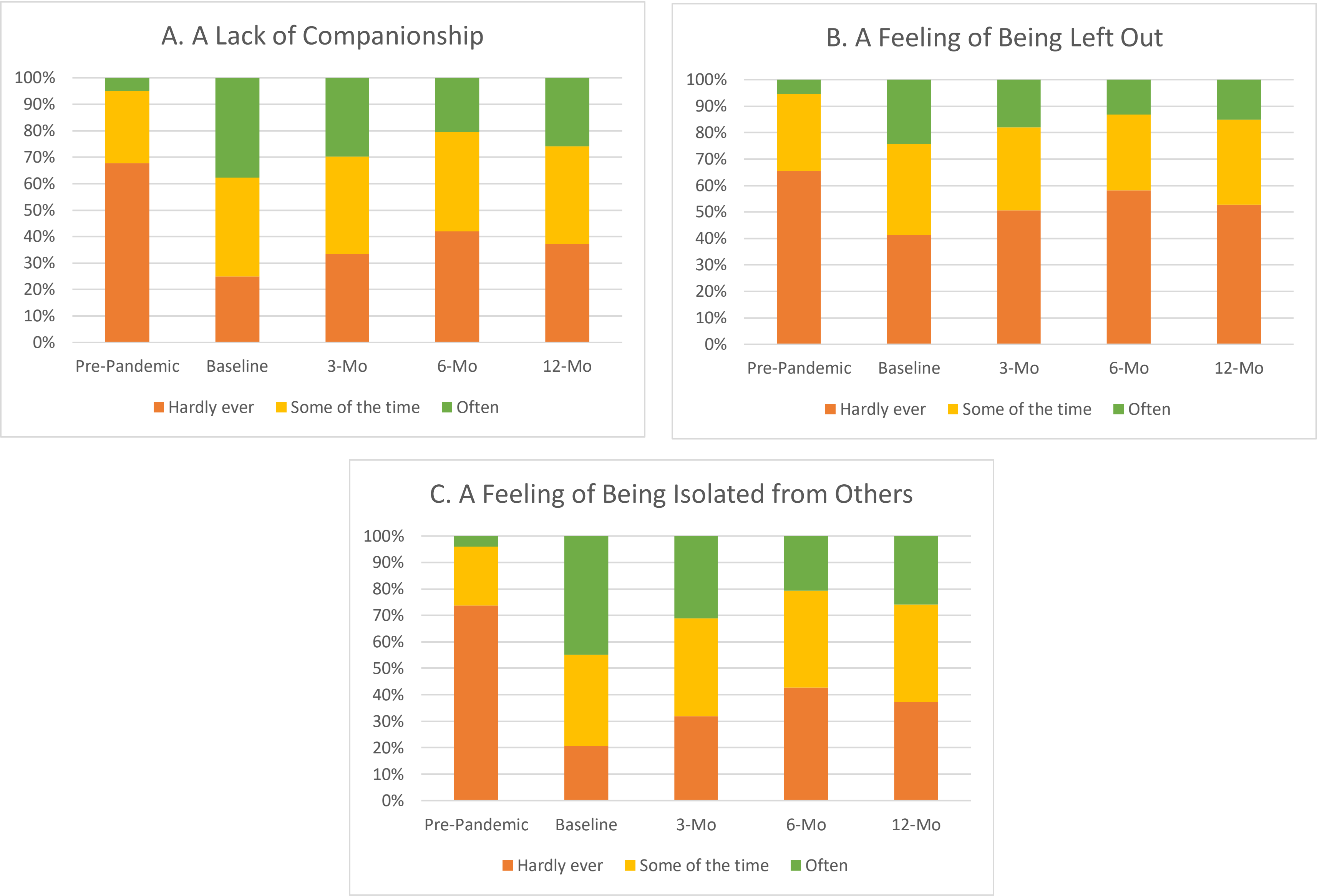

Supplemental Table 1. Perceptions of Work-Related Duties for Completers Dataset*

How much did you experience the following feelings due to your work-related duties? (N (%))							Wilcoxon Test (p-values)				Friedman's Test (p-values)
		**Never**	**Rarely**	**Moderately**	**Quite a bit**	**All the time**	**Pre-Pandemic**	**Baseline**	**3-Mo**	**6-Mo**	
Stress	**Pre-Pandemic**	*14 (1.8)*	*210 (26.4)*	*418 (52.5)*	*137 (17.2)*	*17 (2.1)*					< 0.001
	**Baseline**	*10 (1.3)*	*104 (13.1)*	*286 (35.9)*	*294 (36.9)*	*103 (12.9)*	< 0.001				
	**3-Mo**	*38 (4.7)*	*142 (17.7)*	*307 (38.3)*	*249 (31.1)*	*66 (8.2)*	< 0.001	< 0.001			
	**6-Mo**	*46 (5.8)*	*184 (23.1)*	*294 (37.0)*	*204 (25.7)*	*66 (8.3)*	< 0.001	< 0.001	< 0.001		
	**12-Mo**	*36 (4.5)*	*121 (15.3)*	*286 (36.1)*	*262 (33.0)*	*88 (11.1)*	< 0.001	< 0.001	< 0.001	< 0.001	
Anxiety	**Pre-Pandemic**	*78 (9.8)*	*330 (41.4)*	*291 (36.5)*	*88 (11.0)*	*10 (1.3)*					< 0.001
	**Baseline**	*50 (6.3)*	*177 (22.2)*	*248 (31.1)*	*249 (31.2)*	*74 (9.3)*	< 0.001				
	**3-Mo**	*89 (11.1)*	*230 (28.7)*	*237 (29.6)*	*189 (23.6)*	*56 (7.0)*	< 0.001	< 0.001			
	**6-Mo**	*100 (12.6)*	*267 (33.6)*	*222 (28.0)*	*160 (20.2)*	*44 (5.5)*	< 0.001	< 0.001	< 0.001		
	**12-Mo**	*92 (11.6)*	*188 (23.7)*	*254 (32.0)*	*191 (24.1)*	*69 (8.7)*	< 0.001	< 0.001	< 0.001	< 0.001	
Fatigue	**Pre-Pandemic**	*65 (8.2)*	*338 (42.4)*	*298 (37.4)*	*79 (9.9)*	*17 (2.1)*					< 0.001
	**Baseline**	*48 (6.0)*	*199 (24.9)*	*237 (29.7)*	*218 (27.3)*	*96 (12.0)*	< 0.001				
	**3-Mo**	*82 (10.2)*	*179 (22.3)*	*250 (31.2)*	*224 (27.9)*	*67 (8.4)*	< 0.001	< 0.001			
	**6-Mo**	*114 (14.3)*	*231 (29.1)*	*205 (25.8)*	*183 (23.0)*	*61 (7.7)*	< 0.001	< 0.001	< 0.001		
	**12-Mo**	*82 (10.3)*	*183 (23.1)*	*253 (31.9)*	*183 (23.1)*	*92 (11.6)*	< 0.001	< 0.001	0.003	< 0.001	
Feeling unsafe	**Pre-Pandemic**	*451 (56.6)*	*310 (38.9)*	*27 (3.4)*	*8 (1.0)*	*1 (0.1)*					< 0.001
	**Baseline**	*301 (37.9)*	*248 (31.2)*	*153 (19.3)*	*69 (8.7)*	*24 (3.0)*	< 0.001				
	**3-Mo**	*543 (67.7)*	*202 (25.2)*	*34 (4.2)*	*16 (2.0)*	*5 (0.6)*	< 0.001	< 0.001			
	**6-Mo**	*522 (65.8)*	*188 (23.7)*	*50 (6.3)*	*22 (2.8)*	*11 (1.4)*	0.001	< 0.001	< 0.001		

	**12-Mo**	*364 (46.0)*	*231 (29.2)*	*118 (14.9)*	*57 (7.2)*	*20 (2.5)*	< 0.001	< 0.001	< 0.001	< 0.001	

*'Prefer not to answer' responses were not included in the table and were less than 1% of responses

Supplemental Table 2. Behavioral Outcomes for the Completers Dataset

**Behavioral Outcomes**			**Pre-Pandemic**	**Baseline**	**3-Mo**	**6-Mo**	**12-Mo**
**Physical Activity**	**N (%)**	<30 mins/day	185 (23.2)	297 (37.2)	253 (31.5)	206 (26.0)	277 (35.0)
		>30 mins/day	614 (76.8)	502 (62.8)	549 (68.5)	586 (73.9)	511 (64.6)
	**Wilcoxon (p-value)**	Pre-Pandemic		< 0.001	< 0.001	< 0.001	< 0.001
		Baseline			0.002	< 0.001	< 0.001
		3-Mo				< 0.001	< 0.001
		6-Mo					< 0.001
	**Friedman's (p-value)**		< 0.001				
**Non-work Screen Time**	**N (%)**	<2 hrs/day	482 (60.3)	240 (30.0)	316 (39.4)	355 (44.7)	299 (37.7)
		>2 hrs/day	318 (39.8)	560 (70.0)	486 (60.6)	435 (54.8)	491 (61.9)
	**Wilcoxon (p-value)**	Pre-Pandemic		< 0.001	< 0.001	< 0.001	< 0.001
		Baseline			< 0.001	< 0.001	< 0.001
		3-Mo				< 0.001	0.003
		6-Mo					< 0.001
	**Friedman's (p-value)**		< 0.001				
**Sleep**	**N (%)**	≤7 hours/night	522 (65.2)	415 (51.8)	514 (64.0)	530 (66.8)	517 (65.2)
		>7 hours/night	279 (34.8)	386 (48.2)	288 (35.9)	262 (33.0)	272 (34.3)
	**Wilcoxon (p-value)**	Pre-Pandemic		< 0.001	< 0.001	< 0.001	< 0.001
		Baseline			< 0.001	< 0.001	< 0.001
		3-Mo				< 0.001	< 0.001
		6-Mo					< 0.001
	**Friedman's (p-value)**		< 0.001				
**Alcohol**	**N (%)**	0 drinks/week	254 (31.8)	281 (35.2)	303 (37.7)	262 (33.0)	287 (36.2)
		1-6 drinks/wk	465 (58.2)	384 (48.1)	371 (46.2)	403 (50.8)	395 (49.8)
		7-15 drinks/wk	74 (9.3)	113 (14.2)	97 (12.1)	101 (12.7)	87 (11.0)
		≥16 drinks/wk	6 (0.8)	20 (2.5)	22 (2.7)	19 (2.4)	16 (2.0)
	**Wilcoxon (p-value)**	Pre-Pandemic		< 0.001	< 0.001	< 0.001	< 0.001
		Baseline			< 0.001	< 0.001	< 0.001
		3-Mo				< 0.001	< 0.001
		6-Mo					< 0.001
	**Friedman's (p-value)**		< 0.001				

Supplemental Table 3. COVID-19 Prevention Perceptions for Full Dataset

**How important do you think the following practices are in the prevention of the spread of COVID-19?** (N (%))	**Timepoints**	**Not important**	**Somewhat important**	**Moderately important**	**Very important**
Wearing a mask	**Baseline**	*32 (0.9)*	*71 (2.0)*	*123 (3.5)*	*3265 (93.3)*
	**3-Mo**	*23 (1.3)*	*42 (2.4)*	*70 (4.0)*	*1595 (92.0)*
	**6-Mo**	*26 (1.7)*	*87 (5.8)*	*142 (9.5)*	*1235 (82.6)*
	**12-Mo**	*38 (2.3)*	*58 (3.6)*	*118 (7.2)*	*1421 (86.9)*
Wearing gloves	**Baseline**	*1265 (36.2)*	*1206 (34.5)*	*603 (17.2)*	*398 (11.4)*
	**3-Mo**	*880 (51.0)*	*540 (31.3)*	*188 (10.9)*	*109 (6.3)*
	**6-Mo**	*876 (58.7)*	*408 (27.3)*	*128 (8.6)*	*72 (4.8)*
	**12-Mo**	*1041 (63.7)*	*409 (25.0)*	*120 (7.3)*	*51 (3.1)*
Handwashing	**Baseline**	*14 (0.4)*	*91 (2.6)*	*274 (7.8)*	*3115 (89.1)*
	**3-Mo**	*18 (1.0)*	*93 (5.4)*	*228 (13.2)*	*1392 (80.3)*
	**6-Mo**	*20 (1.3)*	*112 (7.5)*	*221 (14.8)*	*1138 (76.2)*
	**12-Mo**	*32 (2.0)*	*146 (8.9)*	*287 (17.6)*	*1169 (71.5)*
Physical distancing	**Baseline**	*16 (0.5)*	*50 (1.4)*	*157 (4.5)*	*3272 (93.5)*
	**3-Mo**	*8 (0.5)*	*40 (2.3)*	*125 (7.2)*	*1557 (89.9)*
	**6-Mo**	*8 (0.5)*	*68 (4.6)*	*283 (18.9)*	*1134 (75.8)*
	**12-Mo**	*18 (1.1)*	*74 (4.5)*	*331 (20.2)*	*1211 (74.1)*
Disinfecting surfaces	**Baseline**	*139 (4.0)*	*697 (19.9)*	*1066 (30.5)*	*1593 (45.5)*
	**3-Mo**	*213 (12.3)*	*575 (33.2)*	*439 (25.4)*	*502 (29.0)*
	**6-Mo**	*235 (15.8)*	*501 (33.6)*	*386 (25.9)*	*368 (24.7)*
	**12-Mo**	*301 (18.4)*	*531 (32.5)*	*459 (28.1)*	*343 (21.0)*
Getting the COVID-19 vaccine	**Baseline**	*N/A*	*N/A*	*N/A*	*N/A*
	**3-Mo**	*16 (0.9)*	*30 (1.7)*	*65 (3.8)*	*1610 (92.9)*
	**6-Mo**	*11 (0.7)*	*22 (1.5)*	*35 (2.3)*	*1410 (94.4)*
	**12-Mo**	*38 (2.3)*	*25 (1.5)*	*55 (3.4)*	*1505 (92.1)*

Supplemental Table 4. COVID-19-Related Perceptions for Full Dataset

**How much do you agree or disagree with the following statements?** (N (%))		**Strongly Disagree**	**Disagree**	**Neutral**	**Agree**	**Strongly Agree**
Thinking about the coronavirus (COVID-19) makes me feel threatened	**Baseline**	406 (11.9)	747 (21.9)	917 (26.8)	1173 (34.3)	173 (5.1)
	**3-Mo**	221 (12.8)	442 (25.6)	489 (28.3)	520 (30.1)	54 (3.1)
	**6-Mo**	203 (13.7)	381 (25.6)	383 (25.8)	462 (31.1)	57 (3.8)
	**12-Mo**	183 (11.2)	344 (21.1)	420 (25.8)	595 (36.5)	87 (5.3)
I am afraid of the coronavirus (COVID-19)	**Baseline**	240 (7.0)	471 (13.8)	655 (19.2)	1583 (46.3)	467 (13.7)
	**3-Mo**	162 (9.4)	294 (17.0)	355 (20.6)	776 (45.0)	139 (8.1)
	**6-Mo**	145 (9.8)	263 (17.7)	349 (23.5)	608 (40.9)	121 (8.1)
	**12-Mo**	161 (9.9)	301 (18.5)	391 (24.0)	649 (39.8)	127 (7.8)
I am stressed around other people because I worry I'll catch the coronavirus (COVID-19)	**Baseline**	233 (6.8)	459 (13.4)	663 (19.4)	1496 (43.8)	565 (16.5)
	**3-Mo**	170 (9.9)	329 (19.1)	359 (20.8)	699 (40.5)	169 (9.8)
	**6-Mo**	202 (13.6)	419 (28.2)	366 (24.6)	415 (27.9)	84 (5.7)
	**12-Mo**	159 (9.8)	279 (17.1)	353 (21.7)	638 (39.2)	200 (12.3)
Getting the COVID-19 vaccine is very important to me	**Baseline**	N/A	N/A	N/A	N/A	N/A
	**3-Mo**	34 (2.0)	21 (1.2)	80 (4.6)	392 (22.7)	1199 (69.5)
	**6-Mo**	28 (1.9)	14 (1.0)	44 (3.0)	275 (18.5)	1125 (75.7)
	**12-Mo**	45 (2.8)	18 (1.1)	58	306 (18.8)	1202 (73.8)

Supplemental Table 5. Perceptions of COVID-19 Impact on Work for Full Dataset

**Please indicate the extent to which you view the COVID-19 outbreak as having either a positive or negative impact on your work.** (N (%))	**Baseline**	**3-Mo**	**6-Mo**	**12-Mo**
Extremely negative	262 (7.3)	102 (7.3)	81 (5.3)	161 (9.7)
Moderately negative	891 (24.7)	356 (24.7)	263 (17.2)	438 (26.4)
Somewhat negative	1286 (35.7)	643 (35.7)	510 (33.4)	572 (34.4)
No impact	236 (6.5)	123 (6.5)	135 (8.8)	110 (6.6)
Slightly positive	356 (9.9)	213 (9.9)	207 (13.6)	138 (8.3)
Moderately positive	393 (10.9)	216 (10.9)	213 (13.9)	161 (9.7)
Extremely positive	162 (4.5)	99 (4.5)	108 (7.1)	76 (4.6)
Prefer not to answer	21 (0.6)	10 (0.6)	11 (0.7)	6 (0.4)

Supplemental Table 6. Personal COVID-19 Prevention Behaviors for Full Dataset

**In the past month, I have taken the following precautions at my company's workplace** (N (%))		**Never**	**Rarely**	**Sometimes**	**Often**	**Always**
Worn a mask of any type	**Baseline**	*12 (0.6)*	*6 (0.3)*	*18 (0.9)*	*145 (7.2)*	*1837 (91.0)*
	**3-Mo**	*8 (0.9)*	*4 (0.4)*	*9 (1.0)*	*60 (6.6)*	*829 (91.1)*
	**6-Mo**	*138 (15.0)*	*120 (13.0)*	*160 (17.4)*	*161 (17.5)*	*341 (37.1)*
	**12-Mo**	*16 (1.2)*	*21 (2.0)*	*29 (2.1)*	*141 (10.4)*	*1148 (84.7)*
Worn gloves	**Baseline**	*900 (47.1)*	*325 (17.0)*	*353 (18.5)*	*212 (11.1)*	*120 (6.3)*
	**3-Mo**	*494 (57.4)*	*123 (14.3)*	*136 (15.8)*	*77 (8.9)*	*31 (3.6)*
	**6-Mo**	*618 (69.5)*	*104 (11.7)*	*80 (9.0)*	*67 (7.5)*	*20 (2.3)*
	**12-Mo**	*920 (72.3)*	*145 (11.4)*	*125 (9.8)*	*69 (5.4)*	*14 (1.1)*
Washed my hands regularly	**Baseline**	*8 (0.4)*	*13 (0.6)*	*66 (3.3)*	*382 (18.9)*	*1550 (76.8)*
	**3-Mo**	*12 (1.3)*	*5 (0.6)*	*42 (4.6)*	*202 (22.3)*	*646 (71.2)*
	**6-Mo**	*15 (1.6)*	*7 (0.8)*	*46 (5.0)*	*243 (26.2)*	*618 (66.5)*
	**12-Mo**	*6 (0.4)*	*13 (1.0)*	*70 (5.2)*	*360 (26.6)*	*906 (66.9)*
Physically distanced from coworkers or public	**Baseline**	*8 (0.4)*	*19 (1.0)*	*100 (5.0)*	*504 (25.2)*	*1366 (68.4)*
	**3-Mo**	*12 (1.3)*	*7 (0.8)*	*47 (5.2)*	*234 (25.8)*	*607 (66.9)*
	**6-Mo**	*47 (5.1)*	*74 (8.1)*	*210 (22.9)*	*339 (37.0)*	*246 (26.9)*
	**12-Mo**	*13 (1.0)*	*56 (4.1)*	*216 (16.0)*	*592 (43.8)*	*476 (35.2)*
Disinfected surfaces at which I primarily work	**Baseline**	*148 (7.7)*	*186 (9.7)*	*371 (19.4)*	*477 (24.9)*	*732 (38.2)*

	**3-Mo**	*115 (13.5)*	*109 (12.8)*	*180 (21.1)*	*194 (22.7)*	*256 (30.0)*
	**6-Mo**	*193 (21.6)*	*143 (16.0)*	*212 (23.7)*	*193 (21.6)*	*153 (17.1)*
	**12-Mo**	*229 (17.3)*	*237 (17.9)*	*383 (28.9)*	*280 (21.2)*	*195 (14.7)*
Monitored symptoms prior to work (e.g., measuring temperature)	**Baseline**	*143 (7.3)*	*150 (7.6)*	*203 (10.3)*	*249 (12.6)*	*1225 (62.2)*
	**3-Mo**	*97 (11.0)*	*73 (8.3)*	*98 (11.1)*	*123 (13.9)*	*493 (55.8)*
	**6-Mo**	*195 (21.5)*	*119 (13.1)*	*106 (11.7)*	*117 (12.9)*	*372 (40.9)*
	**12-Mo**	*196 (14.7)*	*170 (12.7)*	*212 (16.0)*	*197 (14.8)*	*561 (42.0)*
